# Impact of the COVID-19 Pandemic on Attitudes toward Vaccination: Representative Study of Polish Society

**DOI:** 10.3390/vaccines11061069

**Published:** 2023-06-06

**Authors:** Tomasz Sobierajski, Piotr Rzymski, Monika Wanke-Rytt

**Affiliations:** 1 Center of Sociomedical Research, Faculty of Applied Social Sciences and Resocialization, University of Warsaw, 26/28 Krakowskie Przedmieście Str., 00-927 Warsaw, Poland; 2 Department of Environmental Medicine, Poznan University of Medical Sciences, 60-806 Poznan, Poland; rzymskipiotr@ump.edu.pl; 3 Integrated Science Association (ISA), Universal Scientific Education and Research Network (USERN), 60-806 Poznan, Poland; 4 Department of Pediatrics with Clinical Assessment Unit, Medical University of Warsaw, 63a Żwirki i Wigury Str., 02-091 Warsaw, Poland; monika.wanke@uckwum.pl

**Keywords:** social vaccinology, vaccine hesitancy, social identity theory, self-presentation, SARS-CoV-2

## Abstract

The study explored the association between individuals’ attitudes toward vaccination and their actual vaccination behavior. We also examined the impact of the coronavirus disease 2019 (COVID-19) pandemic and the ongoing vaccination debate on changing attitudes towards vaccination, specifically within different demographic groups. The survey was conducted among a representative sample of Poles (*N* = 805) using computer-assisted web interview (CAWI) technology. As demonstrated, those who identified themselves as strong vaccine supporters were statistically significantly more frequently to be vaccinated with COVID-19 booster doses, to follow a physician’s recommendation on any vaccine without hesitation, and to be strengthened in their confidence in vaccines during the COVID-19 pandemic (*p* < 0.001 for all). However, over half of the responders declared themselves as moderate vaccine supporters/opponents, the groups whose further attitudes are likely to be affected by (mis)communication. Importantly, more than half of moderate vaccine supporters declared that their vaccine confidence was weakened during the COVID-19 pandemic, while 43% were not vaccinated against COVID-19. In addition, the study demonstrated that older and better-educated individuals were more likely to be COVID-19-vaccinated (*p* < 0.001 and *p* = 0.013, respectively). The results of this study imply that, in order to improve vaccine acceptance, it is essential to strengthen public health communication and avoid communication errors conducted during the COVID-19 pandemic.

## 1. Introduction

The coronavirus disease 2019 (COVID-19) pandemic has led to significant morbidity, mortality, and social and economic impacts. It has also been met with an unprecedented response from the scientific community, which engaged, among many things, in the understanding of viral biology and the selection of antigen targets essential for developing vaccine candidates [1,2]. This response, along with the use of more innovative vaccine platforms, such as mRNA and vector technologies, significant funding, and regulatory institutions working at a higher pace, eventually translated into the authorization of the first COVID-19 vaccines at the end of 2020 [3,4]. Their swift and global rollout resulted in significant public health benefits. As estimated, 19.8 million deaths were averted by the COVID-19 vaccination in 2021 alone [5]. Further benefits were also gained by preventing hospitalizations and decreasing the long-term consequences of SARS-CoV-2 infection, known as post-COVID syndrome [6,7,8]. Vaccination also curbed viral transmission and evolution [9,10]. At the beginning of 2023, over 13.1 billion COVID-19 vaccine doses have been administered worldwide, including mono- and bivalent booster doses, introduced in response to waning post-vaccination immunity and the emergence of novel viral lineages [11,12,13].

Despite the availability of COVID-19 vaccines and the evidenced benefits of their administration, vaccine hesitancy and rejection remain significant barriers to decreasing the impacts of SARS-CoV-2 on healthcare systems. It is a complex and multifunctional phenomenon that is influenced by individual, social, and contextual factors, often driven by a lack of trust in the healthcare authorities and government and state institutions, concerns about vaccine safety and efficacy, misinformation, fake news, and intentional anti-vaccine propaganda [14,15,16,17,18,19]. Individuals’ attitudes towards COVID-19 vaccines may differ in relation to not only each particular vaccine technology and manufacturer, but also past experience with vaccines or vaccine doses regarding the adverse effects [20,21,22]. An important component of vaccine hesitancy is constituted by psychosociological predispositions and cultural context [23,24,25,26]. The decision to refuse a vaccine may also involve emotional dedication, which may be challenging to abandon even when confronted with evidence. For example, one study demonstrated that one-third of unvaccinated patients hospitalized with severe COVID-19 will still express vaccine hesitancy or no regrets about vaccine refusal, particularly if they reveal strong conspiracy beliefs [27]. This shows the strength of attachment to such beliefs despite the experience of severe disease, which has also been observed in other contexts; e.g., some individuals living with HIV/AIDS may continue to reveal strong AIDS denialism, believe that HIV is not a causative agent of AIDS, and refuse antiviral treatments [28]. Such attitudes may be highly challenging in various contexts of human health as they represent a general rejection of evidence-based medicine.

The COVID-19 pandemic has been characterized by dynamic changes in the epidemiological situation with subsequent infection waves and the emergence of novel SARS-CoV-2 lineages revealing differences in the clinical course of the disease [29,30]. For a long time, these changes have been closely followed by the media, influencing public perception of the epidemiological threat, depending on the content presentation quality. The unprecedented flood of misinformation has also accompanied this, spread mainly via online social media [31,32,33]. Altogether, these factors could influence the perception of vaccines. Social pressure to vaccinate and the idea of pursuing mandatory COVID-19 vaccination could further polarize society into vaccine supporters and opponents [34,35,36]. The public presence of scientists and healthcare professionals also increased during the COVID-19 pandemic. On the one hand, this could translate into a better understanding of the situation within society because these individuals, and particularly physicians, are role models of pro-health behaviors for society [37]. On the other, it may also lead to confusion or even distrust, particularly when, in the light of novel evidence, the viewpoint had to be dynamically changed or corrected during the pandemic [38]. If such situations are common within the academic community and represent “science at work”, they may not necessarily be well-understood by the general public.

Therefore, exploring the association between the general attitude towards vaccination and the COVID-19 pandemic and trust in healthcare professionals is important. It is also interesting to understand whether individuals with various attitudes differed in their decision to receive the initial COVID-19 vaccine regimen as well as booster doses, which were recommended during the vaccine campaign due to the gradual decrease in serum antibody concentrations and the emergence of viral variants revealing immune-escape [39,40]. Depending on these attitudes, some initially vaccinated individuals may lose interest in booster doses, e.g., due to a breakthrough infection and subsequent decrease of trust in vaccine effectiveness [41].

To this end, the present study investigated the association between individuals’ attitudes toward vaccination and their actual vaccination behavior. We also examined the impact of the COVID-19 pandemic and the ongoing vaccination debate on changing attitudes towards vaccination, specifically within different demographic groups. The statements made by the respondents regarding their vaccination attitudes were considered, as they may lead to actual actions being taken. For this reason, we based the design of our survey and the analysis of the results on the theoretical grid of the idea of self-presentation and social identity theory, which suggests that individuals strive to maintain a consistent image of themselves to others. Declaring one’s intention publicly can strengthen their self-image and dedication to a specific action plan. Based on the theoretical framework of the social identity theory and an analysis of the literature related to people’s attitudes towards COVID-19 vaccination for this study, we hypothesized that people who declare themselves strong vaccine supporters are more likely to vaccinate against COVID-19 and their pro-vaccination attitude has not changed or even strengthened during the COVID-19 pandemic.

## 2. Materials and Methods

### 2.1. Theoretical Framework

The idea of self-presentation refers to the process by which individuals attempt to control or shape the impression others have of them. In psychology, self-presentation regulates emotions, behaviors, and thoughts to create a favorable impression on others. It can involve strategic communication and nonverbal behaviors. In sociology, self-presentation is viewed as a way of constructing a social identity that is consistent with cultural norms and values. Sociologists study how individuals present themselves in different social situations and use various strategies to achieve social goals. In a classic study, Goffman, who called self-presentation “impression management”, argued that individuals engage in various forms of impression management to create a desired image of themselves in the minds of others [42]. The main elements of Goffman’s theory were: (1) front-stage behavior, which refers to the behavior that individuals engage in when they are in the presence of others and are actively trying to manage the impressions they create; (2) backstage behavior, which refers to the behavior that individuals engage in when they are not in the presence of others and are not actively managing their impressions, and this can involve behaviors that are not inconsistent with the front-stage image that individuals present; and (3) dramaturgical analysis, which refers to the idea that individuals engage in a kind of performance when they are in the presence of others, and this performance is shaped by a variety of factors, including the individual’s social status [42].

Social identity theory seeks to explain how individuals form their social identities and how these identities influence behavior and intergroup relations [43]. At its core, SIT posits that individuals derive part of their self-concept from their membership in social groups, and this group membership can be based on a variety of factors. SIT proposes that individuals use social comparison, which involves comparing their group to others, to enhance their self-esteem [44].

### 2.2. Study Design and Population

This study was conducted using a social research framework with a quantitative methodology. Specifically, the survey aimed to assess whether:

- COVID-19 vaccination status in the surveyed individuals was associated with following a physician’s recommendation to receive any vaccine;
- COVID-19 vaccination status was associated with general attitudes toward the vaccines (strongly or moderately supportive or opposing) declared by the studied individuals;
- the COVID-19 pandemic and the ensuing discourse on vaccines affected general confidence in vaccines in the surveyed individuals and whether this effect was modified by their sociodemographic characteristics and declared attitude towards vaccination.

The survey was carried out by the research agency, SW Research, in October 2022. A representative sample of 805 Poles was selected randomly to ensure the best possible representation of the population. The sample was stratified based on demographic characteristics, including sex, age, education, place of residence, and income. The survey was conducted using computer-assisted web interview (CAWI) technology. Each of the drawn participants in the study received a link with information about the study and its purpose, an informed consent form to participate in the study, and a survey questionnaire to fill out on their own with instructions next to the questions.

### 2.3. Questionnaire Design

A questionnaire consisting of 13 closed questions (5 metric and 8 factual) was designed specifically for this study. All questions were of closed character and included a list of answers to choose from. The theoretical framework adopted for the purpose of this study implied one of the main questions of the questionnaire, relating to the respondents’ attitudes towards vaccination. Respondents were asked to classify themselves into one of four groups: strong vaccine proponent, moderate vaccine proponent, moderate vaccine opponent, or strong vaccine opponent. Because of the importance of the vaccination issue, we decided not to allow a middle ground and to create a pro/anti dichotomy, scaling it so that each person could relate to the question to a greater or lesser degree. The questionnaire was validated ad hoc and revised by qualified interdisciplinary experts, then adapted to the study’s technique. The questionnaire was also pilot-tested on a random group of 30 respondents to verify its correctness. The sample from the pilot study was not included in the survey sample on which the results were analyzed.

### 2.4. Statistical Analysis

Data analysis was conducted using IBM SPSS Statistics v. 28.0.1.0 (IBM Corp., Armonk, NY, USA). The results were presented as the total number of respondents (n) and the frequencies of subgroups (%). The chi-squared test was employed to evaluate differences in frequencies expressed by the nominal categorical variables. A *p*-level of <0.05 was considered statistically significant. We used Cohen’s effect size (*d*) to quantify the magnitude of the treatment effect in our study. Based on a confident level of 0.99 and an estimated general population size of approximately 31 million, with an estimated fraction size of 0.5 and a maximum error of 0.05, the minimum required sample size was calculated to be 666 individuals.

### 2.5. Ethical Considerations

The present study followed all ethical rules and regulations of Poland and the European Union concerning social research. The research agency conducting the survey was a member of ESOMAR, providing ethical research implementation and respondent data protection guarantees. The study had a confidential character, and all survey participants gave informed consent to participate in the study, and no individual-level data were used. The study was approved by the Ethics Committee of the Medical University in Warsaw (no. AKBE/77/23).

## 3. Results

### 3.1. Characteristic of Studied Population

The sociodemographic characteristics of the studied group (*N* = 805) are summarized in Table 1. It included a larger percentage of women and was represented by various age groups, mostly individuals with secondary or tertiary education and inhabiting cities of different sizes (Table 1).

One in two respondents (50.6%, *n* = 407) completed the primary regime of COVID-19 vaccination (two doses) and at least one booster dose, 18.3% (*n* = 147) were vaccinated but did not receive a booster dose, and 31.1% (*n* = 251) were unvaccinated. The frequency of vaccinated individuals who received booster doses increased with age and was the highest in the group aged 65 (Table 2). Moreover, it was higher in those with tertiary education. No differences in this regard were noted between women and men and in relation to their place of living. The percentage of unvaccinated individuals in urban areas varied from 24.2 to 30.5%, while in rural areas, it amounted to 35.0% (Table 2).

### 3.2. The Association between Physician Recommendation and Vaccination Attitudes

One in five people (20.6%, *n* = 166) declared that they would vaccinate without hesitation if recommended to do so by a physician. One in four (27.7%, *n* = 223) indicated that they would need to consult first with another healthcare worker, while one in five (21.9%, *n* = 177) would need to review information available online beforehand. A physician’s recommendation would not affect the decision to vaccinate in three out of 10 surveyed individuals (29.7%, *n* = 239).

**Figure 1:**
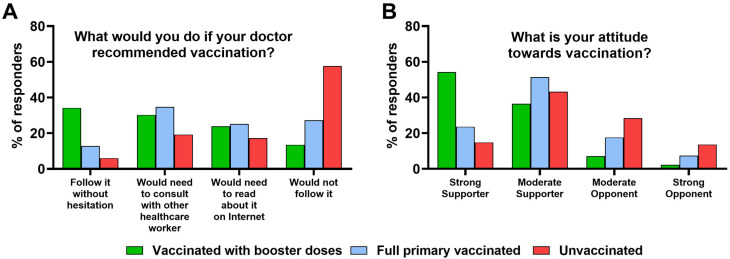
The association between COVID-19 vaccination status and (**A**) whether responder will follow a physician’s recommendation to receive a vaccine and (**B**) responder’s attitude toward vaccinations in the studied group (*N* = 805).

Individuals vaccinated with booster doses declared most frequently that they would follow the vaccination recommendation from their physician without any hesitation (*p* < 0.001). More than half of the responders not vaccinated against COVID-19 (57.6%) indicated that physician recommendation would not affect their decision regarding vaccine uptake (Figure 1A).

### 3.3. The Association between Vaccination Attitudes and COVID-19 Vaccination Status

Of all surveyed individuals, one in three (36.4%, *n* = 293) declared themselves as strong vaccine supporters, four in ten people (41.2%, *n* = 332) as moderate vaccine supporters, one in six people (15.7%, *n* = 126) as moderate vaccine opponents, while 6.7% (*n* = 54) declared themselves as strong vaccine opponents.

The highest frequency of individuals vaccinated with COVID-19 booster doses was found among those who declared themselves as strong vaccine supporters and moderate vaccine supporters (*p* < 0.001) (Figure 1B). However, 17.6% and 7.4% of those who completed the primary COVID-19 vaccination regime indicated represent moderate vaccine opponents and strong vaccine opponents, respectively. Moreover, among strong and moderate vaccine supporters, as many as 14.8% and 43.2% were unvaccinated against COVID-19 (*p* < 0.001) (Figure 1B).

### 3.4. The Association between COVID-19 Pandemic and Vaccination Attitudes

The COVID-19 pandemic and the related discourse on vaccinations strengthened confidence in vaccines among one in five respondents (21.0%, *n* = 169) but weakened it in one in four (25.4%, *n* = 204). Over half of respondents (53.6%, *n* = 432) had no effect of the COVID-19 pandemic and associated discourse on their attitudes toward vaccinations.

Men declared that the COVID-19 pandemic and the ensuing discussion on vaccination strengthened their confidence in vaccines nearly twice as frequently as women (Table 3). Age also had an effect in this regard. In general, the frequency of individuals declaring to experience enhanced confidence in vaccines increased with age, while the frequency of those whose confidence weakened decreased and was the highest among responders aged 18–24 years.

One in four responders with tertiary education declared an increase in confidence in vaccines during the COVID-19 pandemic. Place of living did not significantly differentiate the effect on the perception of vaccines. However, 21.3–25.3% of those living in the city declared their confidence to be strengthened compared to 17.6% in rural areas. In turn, weakened confidence was reported from 22.2% to 30.3% of city inhabitants compared to 25.1% living in rural areas (Table 3).

**Figure 2:**
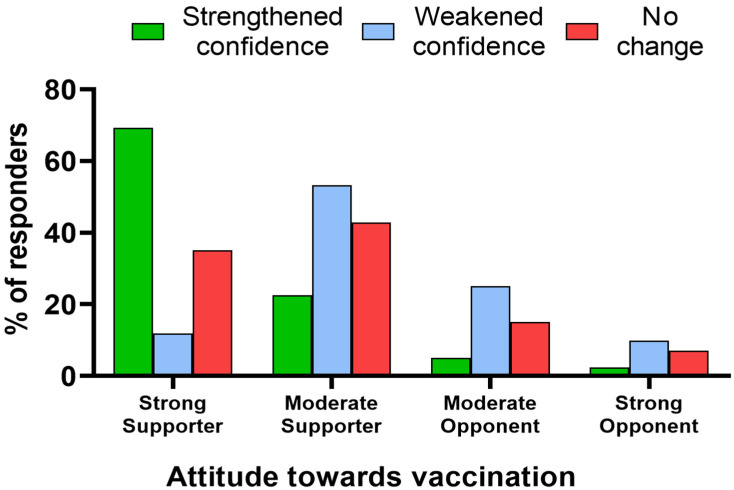
The association between declared attitude toward vaccinations and the effect of the COVID-19 pandemic and the ensuing discourse on vaccines on confidence in them in the studied group (*N* = 805).

Those who declared themselves strong vaccine supporters were most frequent to declare that their confidence in vaccines strengthened during the COVID-19 pandemic (*p* < 0.001) (Figure 2). However, over half of moderate vaccine supporters indicated their confidence was weakened. Only 5.0% of moderate vaccine opponents experienced increased confidence in vaccines during the pandemic, and 2.4% among strong vaccine opponents. These two groups more often declared their confidence to have weakened (*p* < 0.001) (Figure 2).

## 4. Discussion

The present study explored how attitudes toward vaccination influence (non)vaccination behavior. In psychological and sociological theory, declarations are often connected to action through commitment. When individuals publicly declare their intentions or commitments, they are more likely to follow through. It is related to the idea of self-presentation and social identity theory, which suggests that individuals strive to maintain a consistent image of themselves to others [42,43]. Consequently, making a public declaration of intent can help to reinforce an individual’s self-image and commitment to a particular course of action.

Moreover, public declarations can also lead to social pressure and accountability, increasing the likelihood of follow-through on the declared action. In this example, someone who loudly declares themselves as a strong vaccine supporter will feel obligated to vaccinate to avoid appearing inconsistent to others. Similarly, a person who declares themselves as a strong vaccine opponent may translate that declaration into action or inaction, i.e., not vaccinating, because of social pressure and expectations. Declarations can be essential in motivating action and shaping behavior through the reinforcement of self-image and commitment, and through social pressure. In addition, the group may shape attitudes toward vaccination, or the attitudes may result from a desire to please the group.

Our study confirmed the tested hypothesis that people who declare themselves strong vaccine supporters are more likely to vaccinate against COVID-19, and their pro-vaccination attitude has not changed or even strengthened during the COVID-19 pandemic. Polish respondents who identified themselves as pro-vaccination were statistically significantly more likely to vaccinate against COVID-19, including booster doses. In addition, seven out of ten people in this group reported that their pro-vaccination attitude had strengthened during the COVID-19 pandemic.

Our survey results also indicate that more than half of the respondents (56.9%) chose a “moderate” attitude regarding vaccination. Most of this group declared themselves moderate vaccine supporters (41.2% of the total surveyed). Regarding the accepted theoretical framework, one should be very cautious in how they treat the declarations of these individuals. It is because it can be assumed that some of them, while declaring this supportive attitude, disagree in their front-stage behavior and backstage behavior. Among those who declared themselves to be moderate vaccine supporters, more than half declared (53.2%) that the COVID-19 pandemic and the discussion towards vaccination had weakened their confidence in vaccines. This contrasts with only 5% of moderate vaccine opponents who declared an increase in this confidence. Nevertheless, those who chose “moderate” attitudes regarding vaccination are likely the main target of anti-vaccine propaganda, which can further strengthen the self-image of opponents and undermine the confidence among supporters. At present, this can be executed through online social media, which have become the main channel for people to communicate, share content, and access information [45]. The algorithms behind these platforms can amplify the controversial, often scientifically unsupported content, as such a strategy increases user engagement [46]. Moreover, based on the user’s activity and engagement, these algorithms eventually create an “information cocoon” leading to the continuous reinforcement of viewpoints and values [47,48]. Unsurprisingly, the cross-sectional studies report a positive relationship between a reliance on social media and vaccine hesitancy, further supported by the thematic analyses of extracted social media content dominated by anti-vaccine topics [49]. All in all, this leads to the conclusion that effective public health communication must not only encompass different education levels but also include online social media, whose algorithms require redesigning so they could amplify pro-healthy content over the misinformation [50].

However, one should also note that, in the present study, 43% of those who declared themselves to be moderate vaccine supporters and 15% of strong vaccine supporters were not vaccinated against COVID-19. Understanding the exact reasons behind this phenomenon would require further studies. However, it can be hypothesized that this may be due to challenges in science communication that arose during the COVID-19 pandemic. It exposed disparities in health literacy related to low education levels and economic status [16,51]. Individuals who struggle to understand scientific information are more likely not to trust its sources and express vaccine hesitancy. This effect may arise from difficulties in understanding scientific language, jargon, and data presentation [16,52]. As demonstrated in one study, the topic, when presented with a jargon-filled text, engages readers less than when they read about it in clear language [53]. Moreover, the evolving situation of the COVID-19 pandemic and the need to revise the previous views based on the newly emerging scientific evidence could lead to confusion by those not familiar with the scientific process [38]. All of the abovementioned factors could lead to feelings of confusion and the inability to digest health information, leading to alienation from accurate information sources while pointing to sources that offer simple yet inaccurate and misleading answers to complex questions which would further shape decisions not to vaccinate [54]. Ultimately, science and health communication require certain skills and competencies which should be developed by default in scientists and healthcare workers through professional training [55].

The present study also explored the association between the status of COVID-19 vaccination and confidence in physicians’ recommendations regarding the uptake of vaccines. Those who received booster doses declared that they would vaccinate with any vaccine recommended by their physician. Trust is the first of five predictors of the 5C model (confidence, constraints, complacency, calculation, and collective responsibility) developed by Betsch et al. to understand the complex mechanism of social behavior toward vaccination [56]. A lack of trust in the care of health professionals is also associated with a lack of acceptance of vaccination [57,58,59]. In line with this, most of the unvaccinated responders in the present research declared that a physician’s recommendation to receive a vaccine would not affect them. This is worrying because it indicates a significant loss of trust in healthcare professionals and may eventually lead to questioning pro-health attitudes in a broader context instead of just vaccines. Importantly, one study also showed that only two-thirds of unvaccinated patients who required hospitalization due to severe SARS-CoV-2 infection in Poland regretted refusing the COVID-19 vaccine, and one-third declared no will to receive it [27]. Altogether, these observations underline the importance of misinformation in some individuals who are often emotionally attached to their beliefs and not prone to abandon them despite contradictory personal experiences or recommendations from physician.

One should also note that nearly half of the responders declared that, after receiving a physician’s recommendation to receive a particular vaccine, they would need to consult with another healthcare worker or compare it with information available on the Internet. Such an observation indicates that the perception of physicians as role models for decisions on vaccine uptake is, at least in Poland, far from optimal. One of the potential reasons behind this is that their personal attitudes toward vaccination also vary. The study conducted among unvaccinated COVID-19 patients who required hospitalization indicated that sometimes their decision to refuse the COVID-19 vaccine was made under the influence of a physician [27]. The study shows that one-fifth of physicians revealed hesitancy toward COVID-19 vaccination at the beginning of the national vaccination program [60]. Moreover, the vaccination against influenza among Polish physicians is also far from satisfactory, e.g., in the 2016/2017 epidemic season, it amounted only to 32%, with the majority of them being pediatricians and general practitioners, while the lowest vaccination rate was noted among orthopedists and psychiatrists [61]. In summary, more consistency in physicians’ behavior toward their own decisions on vaccinations is required to increase vaccine confidence in the general public. This could be achieved through campaigns increasing awareness of vaccine safety and benefits among this group of healthcare professionals because, as long as they have direct contact with a patient, they may not be well-educated on vaccines, particularly those developed using novel platforms such as mRNA technology [62].

In addition, our study demonstrated a significant relationship between age and COVID-19 vaccination status; the higher the age, the higher the percentage of people vaccinated with the first and booster doses. This is also confirmed by the observations conducted in other countries, e.g., in the US [63,64], Europe [65,66], and the Middle East [67]. Elderly subjects constitute one of the risk groups for severe COVID-19 and the main target of COVID-19 vaccination, including the administration of booster doses due to the generally lower immunogenicity of vaccines in this group [68,69]. As long as the vaccine’s benefits also outweigh its risks for young adults [70], it is pivotal to ensure high vaccine coverage for the elderly to decrease the number of COVID-19-related hospitalizations and deaths [71]. Moreover, our study has shown that individuals with a secondary or tertiary education were more likely to accept the COVID-19 vaccine, which is also in line with other observations [64,67,72]. Better-educated individuals are more likely to reveal higher health awareness and understand the benefits of vaccination. However, one should note that vaccine hesitancy may also affect better-educated groups, e.g., due to a critical-reflexive repertoire that enables one to question what is considered scientific consensus [73]. In early May 2023, the International Health Regulations (2005) Emergency Committee, considering the declining trend of concordance and hospitalization due to COVID-19 and the high population immunity to SARS-CoV-2, recommended moving to the long-term management of the COVID-19 pandemic. The disease is an ongoing health problem and no longer poses an international public health threat [74]. One of the critical factors in preventing the further spread of the COVID-19 pandemic has been widespread vaccination. The best understanding of the public acceptance of vaccination makes it possible to prepare the best vaccine education guidelines for future pandemics.

### Limitations of the Study

We wish to stress the limitations of the present study. Firstly, it provides a single frame of the very long process of the COVID-19 pandemic in Poland and the associated vaccination program implemented in 2021. Indeed, a long-term study would provide an opportunity for a deeper analytical perspective. Nevertheless, this research, conducted on a representative sample at a time when the excitement about the pandemic and COVID-19 vaccination has subsided, provides a valuable picture of the vaccination attitudes of a large European population, which can be an excellent guide for institutions planning to implement vaccination programs as part of a health policy focused on prevention in child and adult health. The second limitation includes the correlative nature of the study. Although some individuals may accept or reject COVID-19 vaccination due to their pre-pandemic attitude toward vaccination, some may also become vaccine supporters or opponents in response to COVID-19 vaccination and its effects, and this relationship may depend on numerous individuals factors, e.g., adverse events or breakthrough infection. 

## 5. Conclusions

Our study showed a solid relationship between the declared attitude towards vaccination and the actual decision to vaccinate. In particular, those who identified themselves as strong vaccine supporters revealed pro-vaccination behavior, and were more likely to receive booster COVID-19 doses and follow a physician’s recommendation to receive any vaccine. However, over half of the responders represented a “gray zone” of moderate supporters or opponents, the groups whose future attitudes are most likely to be affected by further vaccine (mis)communication. Therefore, it is pivotal to strengthen public health communication. Improving it requires, among many things, engaging more healthcare workers and scientists in the process, improving their skills and competencies through specific training, and incorporating online social media in the process. It is also pivotal to avoid communication errors conducted during the COVID-19 pandemic, which could translate into weakened confidence in vaccines as evidenced in our study.

## Figures and Tables

**Table 1:** Sociodemographic characteristics of the study population (*N* = 805).

	*n*	%
**Sex**		
**Female**	428	53.1
**Male**	377	46.9
**Age**		
**18–24**	100	12.4
**25–34**	153	19.0
**35–49**	195	24.3
**50–64**	231	28.7
**65 and more**	126	15.7
**Education**		
**Primary**	29	3.6
**Vocational**	59	7.3
**Secondary**	411	51.1
**Tertiary**	306	38.0
**Place of living**		
**Rural area**	323	40.1
**City < 20 k**	94	11.7
**City 20–99 k**	154	19.1
**City 100–199 k**	66	8.2
**City 200–499 k**	73	9.0
**City > 500 k**	95	11.8

**Table 2:** Status of COVID-19 vaccination status in the surveyed group (*N* = 805) according to sociodemographic categories.

	Primary Regime and Booster Doses	Primary Regime without Booster Dose	Unvaccinated	*p*-Value	Cohen’s *d*
*n* (%)					
**Sex**					
**Female**	202 (47.3)	83 (19.4)	142 (33.3)	0.135	0.138
**Male**	205 (54.4)	64 (17.0)	108 (28.6)		
**Age**					
**18–24**	34 (34.0)	21 (21.0)	45 (45.0)	<0.001	0.601
**25–34**	51 (33.3)	40 (26.1)	62 (40.5)		
**35–49**	87 (44.4)	45 (23.0)	64 (32.7)		
**50–64**	138 (59.7)	31 (13.4)	62 (26.8)		
**65 and more**	97 (77.0)	11 (8.7)	18 (14.3)		
**Education**					
**Primary**	11 (37.9)	4 (13.8)	14 (48.3)	0.013	0.039
**Vocational**	27 (46.6)	10 (17.2)	21 (36.2)		
**Secondary**	193 (47.0)	74 (18.0)	144 (35.0)		
**Tertiary**	175 (57.2)	59 (19.3)	72 (23.5)		
**Place of living**					
**Rural area**	147 (45.5)	63 (19.5)	113 (35.0)	0.406	0.224
**City < 20 k**	45 (47.4)	21 (22.1)	29 (30.5)		
**City 20–99 k**	80 (51.9)	29 (18.8)	45 (29.2)		
**City 100–199 k**	40 (60.6)	10 (15.2)	16 (24.2)		
**City 200–499 k**	42 (57.5)	11 (15.1)	20 (27.4)		
**City > 500 k**	53 (55.8)	13 (13.7)	29 (30.5)		

**Table 3:** The effect of the COVID-19 pandemic and associated discourse on vaccination on attitudes toward vaccines in the surveyed group (*N* = 805).

	Strengthened the Confidence in Vaccines	Weakened the Confidence in Vaccines	Had no Effect on the Attitude toward Vaccines	*p*-Value	Cohen’s *d*
*n* (%)					
**Sex**					
**Female**	66 (15.4)	113 (26.4)	249 (58.2)	<0.001	0.329
**Male**	103 (27.2)	92 (24.3)	183 (48.4)		
**Age**					
**18–24**	11 (11.0)	37 (37.0)	52 (52.0)	<0.001	0.700
**25–34**	21 (13.8)	50 (32.9)	81 (53.3)		
**35–49**	32 (16.4)	48 (24.6)	115 (59.0)		
**50–64**	58 (25.1)	52 (22.5)	121 (52.4)		
**65 and more**	48 (38.1)	17 (13.5)	61 (48.4)		
**Education**					
**Primary**	2 (6.9)	7 (24.1)	20 (69.0)	0.042	0.173
**Vocational**	9 (15.5)	9 (15.5)	40 (69.0)		
**Secondary**	82 (20.0)	113 (27.5)	216 (52.6)		
**Tertiary**	75 (24.6)	74 (24.3)	156 (51.1)		
**Place of living**					
**Rural area**	57 (17.6)	81 (25.1)	185 (57.3)	0.674	0.115
**City < 20 k**	20 (21.3)	27 (28.7)	47 (50.0)		
**City 20–99 k**	39 (25.3)	36 (23.4)	79 (51.3)		
**City 100–199 k**	15 (22.7)	20 (30.3)	31 (47.0)		
**City 200–499 k**	14 (19.4)	16 (22.2)	42 (58.3)		
**City > 500 k**	23 (24.2)	24 (25.3)	48 (50.5)		

## Data Availability

The data presented in this study are available upon request from the corresponding author.
